# Fracture Types Influence the Likelihood of Lower Urinary Tract Injuries in Patients with Pelvic Fractures

**DOI:** 10.3390/jcm12082967

**Published:** 2023-04-19

**Authors:** Xuehui Zhao, Shun Lu, Bingzhi Wang, Xiaofeng Zhou, Fanxiao Liu, Weicheng Xu, Dongsheng Zhou, Lianxin Li, Jinlei Dong

**Affiliations:** Department of Orthopaedics Surgery, Shandong Provincial Hospital Affiliated to Shandong First Medical University, Jinan 250021, China

**Keywords:** pelvic fracture, lower urinary tract injuries, Tile classification, Young–Burgess classification

## Abstract

Background: The combination of pelvic fractures with lower urinary tract injuries (LUTIs) is a severe traumatic injury. This study was performed to determine the relationship between LUTIs and pelvic fracture types. Methods: Patients who sustained pelvic fractures combined with LUTIs between 1 January 2018 and 1 January 2022 in our institution were retrospectively analyzed. The patients’ demographics, mechanism of injury, presence of open pelvic fractures, types of pelvic fractures, patterns of LUTIs, and early complications were analyzed. The association between pelvic fracture types and the identified LUTIs was statistically analyzed. Results: This study involved 54 patients diagnosed with pelvic fractures combined with LUTIs. The overall incidence of pelvic fractures combined with LUTIs was 7.7% (*n* = 54/698). All patients had unstable pelvic fractures. The male:female ratio was approximately 2.4:1.0. The incidence of LUTIs was higher in men than women with pelvic fractures (9.1% vs. 4.4%). Bladder injuries occurred at roughly equal rates in men and women (4.5% vs. 4.4%, *p* = 0.966), but urethral injuries were more frequent in men (6.1% vs. 0.5%, *p* = 0.001). The most common pelvic injury pattern was a type C fracture according to the Tile classification and a vertical-shear-type fracture according to the Young–Burgess classification. The Young–Burgess fracture classification was related to the severity of bladder injury in men (*p* = 0.037). There was no significant difference in bladder injury according to the two classifications among women (*p* = 0.524 vs. *p* = 1.000) or among the entire cohort (*p* = 0.454 vs. *p* = 0.342). Conclusions: Men and women are equally likely to sustain a bladder injury, but a urethral injury with pelvic fracture is more frequent in men. LUTIs tend to be accompanied by unstable pelvic fractures. It is imperative to be vigilant for potential bladder injury when men sustain vertical-shear-type pelvic fractures.

## 1. Introduction

Pelvic fractures are severe traumatic injuries with high mortality and disability rates, and they often impose a substantial burden on patients and society [1,2,3]. Pelvic fractures are usually present in combination with polytrauma involving the brain, thorax, abdomen, spine, extremities, or other regions [4,5,6]. Anatomically, the bony pelvis is a funnel-like structure acting as a protective shield for the structures within it. These structures include the bladder and the urethra, which constitute the lower urinary tract. Because of their anatomical location, the internal structures of the pelvis are at a greatly increased risk of damage in the event of a fracture caused by a force of high kinetic energy. Therefore, the lower urinary tract is highly susceptible to injury as a result of a displaced pelvic fracture [7,8]. Lower urinary tract injuries (LUTIs) are among the most severe injuries associated with pelvic fractures [9,10].

The incidence of pelvic fractures combined with LUTIs is approximately 4% to 5% [6,11]. Although the incidence of LUTIs associated with pelvic fractures is relatively low, their short-term or long-term complications, such as peritonitis, urinary fistulae, urinary tract infection, urethral stricture, and sexual dysfunction, may be clinically significant [12]. Complex pelvic fractures often put patients at risk of severe bleeding, and rescue-focused therapy in the initial stage of treatment might neglect concomitant comorbidities, such as genitourinary and lower gastrointestinal injuries [8,13,14]. Prompt recognition and management of these injuries is, therefore, of paramount importance for these patients.

Research has indicated that vertical shear (VS)-type pelvic fracture and a compromised pubic symphysis are related to a higher severity of vaginal injury [15]. This finding appears to be of diagnostic and predictive significance in patients with concomitant injuries after pelvic fractures. However, relatively little research has focused on the relationships between specific LUTIs and pelvic fracture types. Two decades ago, Rie Aihara investigated whether any additional factors can serve as markers for rectal injuries and LUTIs, shedding light on the potential relationship between pelvic fracture locations and these injuries [16]. In a recent retrospective cohort study of the relationship between pelvic fracture classifications and LUTIs, a higher incidence of LUTIs was found in patients with unstable pelvic fractures (Tile types B and C) according to the Tile classification [17]. However, after reviewing the existing literature we found that few patients in previous studies were systematically classified by sex, specific organ injury, and different pelvic fracture classifications. Additionally, scholars to date have only explored the potential relationship between the fracture location or a single fracture classification system and LUTIs. No comprehensive analyses have focused on the relationship between bladder/urethra injury and pelvic fractures based on the Tile classification or Young–Burgess classification in men or women. Our research team has conducted serial studies of other concomitant injuries, such as vaginal injuries, rectal injuries, and sciatic nerve injuries, after pelvic fractures and analyses of their corresponding risk factors [15,18,19,20,21]; however, population-based cohort studies of pelvic fractures combined with LUTIs have not been carried out.

The present study was performed to investigate the incidence of LUTIs in patients with pelvic fractures and to explore the association between LUTIs and pelvic fractures according to the Tile classification [22] and Young–Burgess classification [23].

## 2. Materials and Methods

### 2.1. Sample Collection

All patients who presented with pelvic fractures from 1 January 2018 to 1 January 2022 were identified in the medical record information database of our institution. The data of 54 patients with International Classification of Disease version 9 diagnostic codes for traumatic fractures of the pelvis (808.0–808.9) and traumatic injuries to the bladder or urethra (867.0–867.1) were extracted and evaluated. This retrospective study was approved by the ethics committee of our institution.

The inclusion criteria were pelvic fractures combined with LUTIs, an obvious history of trauma, blunt traumatic injury, and complete medical record and imaging data. The exclusion criteria were pelvic fractures combined with upper urinary tract injuries, previous iatrogenic injury, pathologic fractures, congenital pelvic malformations, congenital lower urinary tract malformations, a history of bladder urothelial carcinoma or chronic inflammation, and penetrating injury.

### 2.2. Diagnostic Methodologies

The diagnosis of pelvic fractures combined with LUTIs is comprehensive and is often dependent upon a history of trauma, clinical signs and symptoms, physical examination findings, and imaging examination results. Pelvic fractures should be clinically suspected if patients have a history of sustaining a high-energy impact, such as a road traffic accident or falling from a height, and are exhibiting symptoms or signs, such as pelvic pain, a perineal hematoma, shock, or altered consciousness. Imaging examinations (pelvic X-ray, computed tomography (CT), or CT with three-dimensional bone reconstruction) can establish the final diagnosis. In the present study, these imaging data of all patients were reviewed at the time of injury by the attending general surgeon, orthopedic surgeon, and urologic surgeon. The types and severity of the fractures based on the Tile classification and Young–Burgess classification were determined by three experienced orthopedic surgeons. Any disagreement was resolved through consensus. Likewise, the diagnosis of LUTIs was based on a similar diagnostic workflow. Clinical signs of bladder injury were often present: gross hematuria, suprapubic or abdominal pain or tenderness, dysuria, or even peritoneal irritation. Ultrasonography, CT, and cystography could be used to diagnose bladder injury. In particular, the presence of urethral injury with the following clinical features was also noted: blood at the urethral meatus, difficulty/inability to void, urinary retention, and perineal/scrotal hematoma. CT and cystourethrography were helpful for the diagnosis of urethral injury. The severity of LUTIs was jointly determined by urological surgeons and imaging specialists.

### 2.3. Management

**Figure 1:**
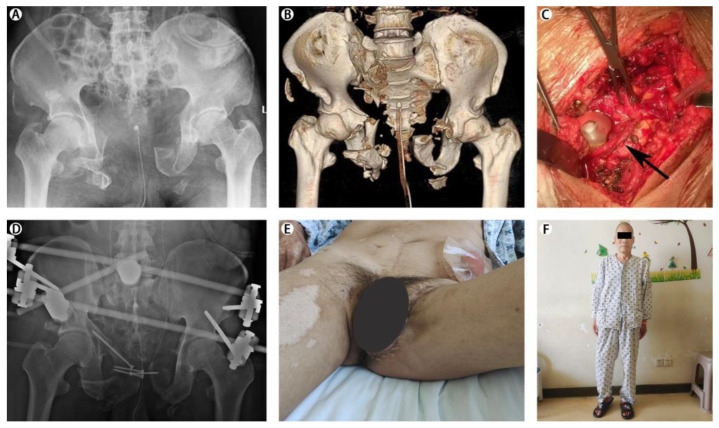
This figure shows a typical case we treated. A 59 year old man was hit by a truck and sustained pelvic fracture combined with lower urinary tract injuries: (**A**,**B**) imaging and examination revealed a Tile type C (vertical shear) pelvic fracture with symphysis pubis and sacroiliac joint severe disruption; (**C**) emergency exploratory laparotomy revealed bladder and posterior urethra rupture; (**D**) the patient underwent anterior pelvic external fixation combined with screw internal fixation and primary repair; (**E**) open perineal wound and surgical incisions healed well; (**F**) he could stand and walk normally at the time of follow-up.

All patients with pelvic fractures and LUTIs were evaluated and resuscitated according to Advanced Trauma Life Support^®^ protocols [24,25]. Fluid resuscitation is the most important aspect in the management of critically ill patients with hemodynamically unstable pelvic fractures. External fixation was performed in patients with open pelvic fractures or severely unstable pelvic fractures. Open reduction and internal fixation was the preferred surgical procedure for pelvic fractures without gross contamination in the fracture region. Bladder contusion and urethral contusion were often conservatively managed. Exploratory laparotomy and primary bladder repair were required for intraperitoneal bladder rupture as shown by a contrast cystogram. Extraperitoneal bladder injuries were often successfully managed by Foley catheter drainage alone, but when the abdomen was explored for associated injuries, extraperitoneal bladder ruptures were repaired at the same time. The preferred method of managing patients with urethral disruption was suprapubic cystostomy drainage with delayed urethroplasty 3 months after pelvic fixation. All patients underwent procedures performed by the same surgical team. The following is a typical case that was treated at our institution (Figure 1).

### 2.4. Clinical Information

A retrospective chart review was performed for all patients with pelvic fractures combined with LUTIs. We predeveloped Excel tables for data collection and selected study indicators, including demographics (age and sex), mechanism of injury, hospital length of stay, injury scenarios, descriptive measures, such as the injury severity score (ISS) and abbreviated injury scale (AIS) score, types of pelvic fractures based on the Tile classification and Young–Burgess classification, patterns of LUTIs, clinical signs and symptoms, whether the fractures were open or closed, management of the pelvic fractures with LUTIs, and treatment outcomes. In addition, the following primary inpatient complications were reviewed: pneumonia, deep vein thrombosis, wound infection, bacteremia, urinary tract infection, acute respiratory distress syndrome, multiple organ dysfunction syndrome, acute kidney injury, shock, and death. The demographic information and fracture types of each patient were reviewed twice by two researchers. If there was a disagreement, another senior physician checked to ensure the consistency and accuracy of the data. 

### 2.5. Statistical Analysis

The data are presented as the proportion, mean ± standard deviation, and range, as appropriate. The categorical variables were analyzed using the chi-square test or Fisher’s exact test, and the continuous variables were analyzed with the Student’s *t*-test. All statistical analyses were performed using SPSS software version 25.0 (IBM Corp., Armonk, NY, USA). A *p*-value of <0.05 was considered statistically significant.

## 3. Results

### 3.1. General Information and Incidences 

In total, 54 patients diagnosed with a combination of pelvic fractures and LUTIs were identified from 698 patients with pelvic fractures referred during the 4 years study period. The patients comprised 45 (83.3%) men and 9 (16.7%) women, with a mean age of 42.81 ± 14.41 years, mean hospital length of stay of 35.37 ± 22.57 days, mean ISS of 35.15 ± 14.55, and mean AIS score of 25.24 ± 12.17. The main trauma mechanisms were road traffic accidents and falling from a height (*n* = 35/54, 64.8%). Ten (18.5%) of the fifty-four patients had open fractures. No patients with fractures in the orthogeriatric population from standing height were found with associated LUTIs.

The total incidence of LUTIs was 7.7% (*n* = 54/698). Among the 54 patients included in the study, 23 had an LUTI of the bladder, 23 had an LUTI of the urethra, and 8 had coexisting LUTIs of both the bladder and urethra. Twenty-two of the thirty-one patients with bladder injuries were male; the remaining nine patients were female. Thirty of the thirty-one patients with urethral injuries were male; only one female patient sustained a urethral injury, and this occurred in combination with catastrophic bladder rupture. The patients’ demographic information is listed in Table 1.

### 3.2. Pelvic Fractures According to Two Classification Systems

According to the Tile classification, the highest proportions of pelvic fractures among all patients were type C1 (*n* = 16/54, 29.6%) and type B2 (*n* = 12/54, 22.2%). Type C1 pelvic fracture was the most common injury pattern in men (*n* = 14/45, 31.1%), followed by types B2 (*n* = 10/45, 22.2%) and C2 (*n* = 9/45, 20.0%). Among women, types B2, C1, and C3 occurred in two patients each.

**Figure 2:**
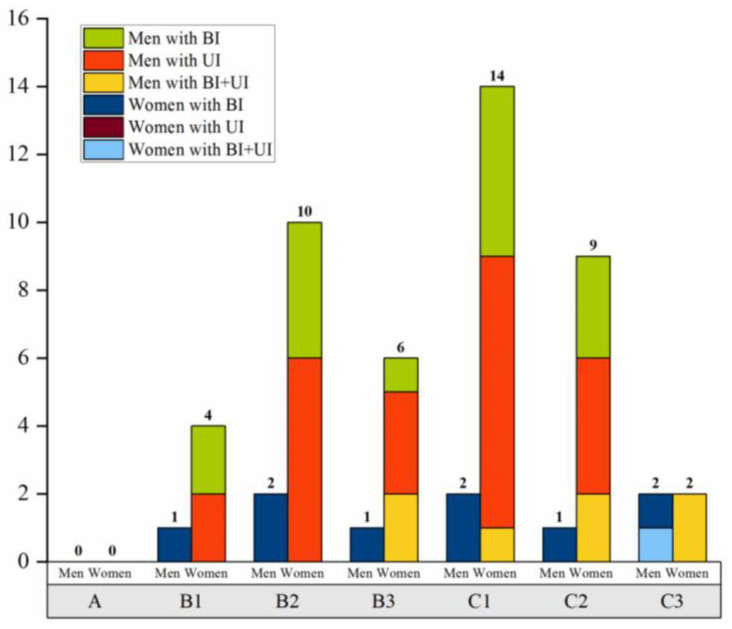
Distribution of LUTIs according to the Tile classification. BI, bladder injury; UI, urethra injury.

**Figure 3:**
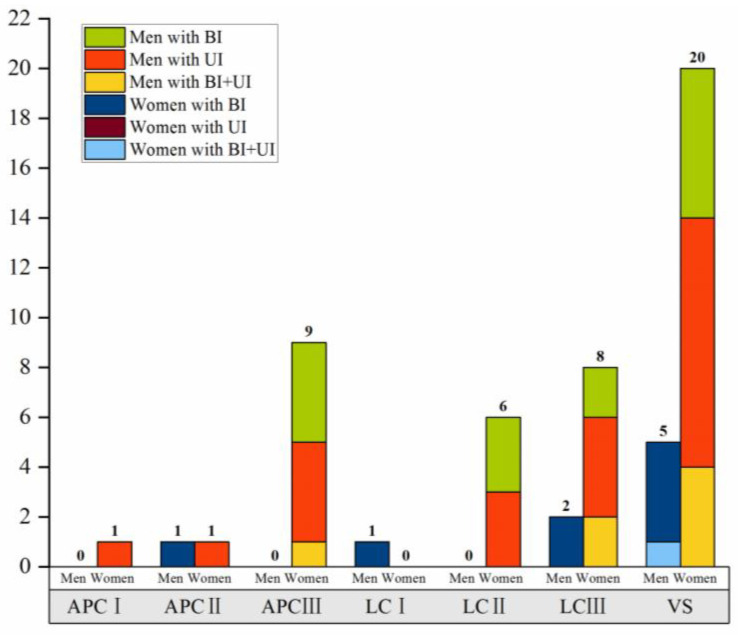
Distribution of LUTIs according to the Young–Burgess classification. BI, bladder injury; UI, urethra injury; APC, anteroposterior compression; LC, lateral compression; VS, vertical shear.

According to the Young–Burgess classification, the highest proportions of pelvic fractures among all patients were type VS (*n* = 25/54, 46.3%) and type lateral compression III (LCIII) (*n* = 10/54, 18.5%). Type VS pelvic fracture was the most common type in men (*n* = 20/45, 44.4%), followed by types anteroposterior compression III (APCIII) (*n* = 9/45, 20.0%) and LCIII (*n* = 8/45, 17.8%). In women, type VS pelvic fracture was also the most frequent (*n* = 5/9, 55.6%), followed by LCIII (*n* = 2/9, 22.2%). Detailed information on the distribution of pelvic fracture types in all patients with LUTIs according to the Tile classification and Young–Burgess classification is shown in Table 2 and Figure 2 and Figure 3.

### 3.3. Distribution Difference of Pelvic Fracture Classifications in Patients with LUTIs

The distribution difference of the types of pelvic fractures among patterns of LUTIs in the overall study population and in men and women separately were evaluated according to the Tile classification and Young–Burgess classification. However, there were no statistically significant differences between the fracture type groups in the specific patterns of LUTIs (Table 2).

Next, we analyzed the distribution difference between types of pelvic fractures and particular organ injuries in men and women separately. Table 3, Table 4, Table 5 and Table 6 show the outcomes of the statistical analyses. The statistical results showed a significant difference in the Young–Burgess classification and bladder injury, and the VS-type pelvic fracture was associated with a higher severity of bladder injury in men (*p* = 0.037) (Table 5). Bladder injuries were not associated with the Tile fracture classification in men according to our classification method (*p* = 0.070) (Table 5). Similarly, in women, there was no significant difference in bladder injury between the two classification systems (*p* = 0.524 vs. *p* = 1.000) (Table 5). No significant difference was found between the types of pelvic fractures and urethral injury in men (*p* > 0.05) (Table 6). Because only one female patient sustaining a urethral injury was included in this study, the data could not be analyzed statistically.

### 3.4. Complications and Mortality

A total of 49 (90.7%) patients developed at least one complication. The most common complication was shock (46.3%), followed by pneumonia (44.4%), deep vein thrombosis (42.6%), wound infection (33.3%), acute respiratory distress syndrome (24.1%), and acute kidney injury (14.8%). Of all 54 patients who were admitted to our hospital, three (5.6%) died during their hospital stay (Table 1). A 71-year-old woman with brain trauma died of an acute cerebrovascular accident during attempted defecation 1 week after surgery, and a 63-year-old man with a severe open pelvic fracture died of a severe infection caused by a multidrug-resistant organism. In addition, a 22-year-old woman with severe systemic polytrauma (ISS of 66) died of multiple organ failure 1 month after undergoing hemipelvectomy; she was the only female patient with urethral rupture among all patients in this study.

## 4. Discussion

According to the current literature, the incidence of LUTIs in patients with pelvic fractures is approximately 4% to 5%. Corró et al. [17] performed a retrospective review and identified 25 (4.1%) patients with LUTIs among 614 patients with pelvic fractures treated at their center from 2007 to 2015. Johnsen et al. [26] reported that 233 (4.2%) of 5518 patients admitted to a level I trauma center from 2000 to 2014 had LUTIs associated with pelvic fractures. In a large analysis performed by Bjurlin et al. [11] using the National Trauma Data Bank, 1444 (4.6%) LUTIs were reported among 31,380 patients with pelvic fractures. In the present study, the overall incidence of pelvic fractures combined with LUTIs was 7.7%, with the same overall incidence of bladder injuries and urinary tract injuries. We speculate that the higher incidence rate in our study is because our hospital is a large tertiary hospital, as well as a regional trauma center. As a result, many patients with severe trauma are transferred from other lower-level hospitals to our hospital for treatment, undoubtedly increasing the incidence of LUTIs in patients with pelvic fractures in our institution.

Similarly, in the present study, we found that men contributed to a higher proportion of pelvic fracture cases and were more likely to sustain LUTIs associated with pelvic fractures; this finding is similar to that reported by Bjurlin et al. [11]. According to our data, men and women were equally likely to sustain a bladder injury after having sustained pelvic fractures (4.5% vs. 4.4%, *p* = 0.966); however, urethral injuries were more common in men than in women (6.1% vs. 0.5%, *p* = 0.001), and this is consistent with the findings of Johnsen et al. [26] and Bjurlin et al. [11]. We speculate that this occurred because the anatomy of the genitourinary system is significantly different between men and women. Pomian et al. [27] studied the urethral length of 927 women in a prospective study from 2013 to 2017 and found that the mean length of the female urethra was 30.1 ± 4.2 mm (range: 19–45 mm). Kohler [28] collected the data of 109 men and reported that the mean length of the male urethra was 22.3 ± 2.4 cm. The female urethra is shorter and more mobile than the male urethra; anatomically, it is almost completely protected by the pubic bone and pelvic floor muscles surrounding the urethra [29,30]. This might be the reason for the low incidence rate of urethral injury in women [7].

The most common mechanism of injury in patients with LUTIs associated with pelvic fractures in this study was road traffic accidents (38.9%). Falling from a height (25.9%) was another important mechanism. This strongly suggests that LUTIs associated with pelvic fractures are the result of high-energy blunt force trauma [31]. However, another review by Samir et al. [32] focusing on the relationship of the mechanism of force delivery and the magnitude and direction of the impact force on the pattern of associated organ injuries after pelvic fractures showed that the two most common mechanisms of injury were motor vehicle accidents and being struck as a pedestrian (57.4% and 17.8%, respectively). The authors further analyzed the relationship between the mechanism of injury and pelvic fracture type, and the results are discussed below.

When comparing the pattern of pelvic fractures among the 54 patients in the present study, we found that LUTIs occurred more often in association with certain types of pelvic fractures. We found that the most common pelvic injury pattern according to the Tile classification was type C fracture (*n* = 30, 55.6%); all of the remaining 24 patients sustained type B fractures. No patients had type A fractures. Our results are in concordance with the findings reported by Corró et al. [17]. These findings seem to validate the report by Corró et al. [17] that the highest incidence of LUTIs occurs in association with unstable pelvic fractures (Tile types B and C). In the Young–Burgess system, VS-type fractures accounted for the largest number of pelvic fractures (*n* = 25, 46.3%). Next, when we analyzed the types of fractures associated with bladder injury and urethral injury separately, we found that the VS type was the most common for both, while the proportion of types B and C was approximate. Samir et al. [32] reported that the most common and second most common types of pelvic fractures were LC and APC, whereas VS injuries accounted for only 4.7%. We consider that these differences might have arisen from above-described mechanism of the pelvic fractures. Samir et al. [32] noted that the cause of the pelvic fracture resulted in certain important differences in the pattern of incidence of the types of pelvic fractures and their degrees of severity as classified above. The examination of the authors’ data demonstrated that the fracture type with the highest incidence was LC among motor vehicle injuries and APC among crush injuries; the VS type was much more common in patients who had fallen from a height [32]. Likewise, Andrich [33] noted that VS fractures were typically sustained by individuals falling from a height. As might be expected based on the mechanism of Injury, the higher proportion of injuries due to the fact of falling from a height was likely responsible for the higher incidence of VS-type fractures in our study.

Samir et al. [32] reported that the type of mechanical force and severity of the pelvic fracture are the keys to the expected organ injury pattern. Pengyu et al. [15] proposed the hypothesis that the pelvic fracture pattern may influence the occurrence of vaginal injury, and the final results confirmed that VS-type fractures and a compromised pubic symphysis were associated with more severe vaginal injury. Notably, although Andrich et al. [33] suggested that the pelvic fracture pattern alone did not predict the presence of an LUTI, they observed that among men with complete urethral disruption, combined or complex LUTIs were only found among those with type C (VS) fractures. Corró [17] found a higher incidence of LUTIs in patients with unstable pelvic ring fractures (Tile types B and C) and reported that type C3 pelvic ring fractures were more frequently associated with LUTIs. This appears to suggest that VS-type fractures are associated with more severe LUTIs. We evaluated the severity of bladder injury and urethral injury in men and women associated with different fracture patterns separately, and the results showed that only bladder injury in men was correlated with the Young–Burgess fracture classification. Specifically, we found that VS-type fractures were associated with more severe bladder injury in men (*p* = 0.037) (Table 2). However, we were unable to validate this finding in women, because the number of women in our study was too small. This result suggests that the Young–Burgess system has important implications in predicting bladder injury in men. However, Corró et al. [17] noted that bladder injuries were not associated with any specific type of instability, but it should be stressed that they focused solely on the Tile classification. Interestingly, Samir et al. [32] reported a greater association of urethral and bladder injuries with APC fractures. Aihara et al. [16] concluded that widening of the pubic symphysis and sacroiliac joint would increase the risk of bladder injury. Mechanisms of bladder injury in patients with pelvic trauma include direct penetration by bony fragments, compression, and shearing forces transmitted through ligamentous attachments. Because the precise mechanism of bladder injury varies, the pattern of injury is difficult to accurately predict. As a result, further studies are required to elucidate the possible mechanism of injury. According to the Tile classification, pelvic fractures are categorized into type A (inherently stable), type B (rotationally unstable but vertically stable), and type C (rotationally and vertically unstable); while the Young–Burgess classification includes anterior–posterior compression (APC), lateral compression (LC), vertical shear (VS) injuries, and combined mechanisms (CM). The pubic symphysis and sacroiliac joint readily become separated and displaced when the pelvis is subjected to VS forces [34]; therefore, we hypothesize that VS fractures are more likely to result in bladder injury. We conclude from our findings that clinicians need to be alert to the presence of bladder injury in men with VS-type pelvic fractures.

Because the forces involved in pelvic fractures are high-energy forces, LUTIs combined with pelvic fractures tend to be associated with multiple and life-threatening injuries. As a result, attention to resuscitation tends to predominate in the initial management of these patients [13,25,35]. Prompt fracture reduction and stabilization is the primary goal of orthopedic operations [13,36]. However, selection of the fixation technique for pelvic fractures should be determined on a patient-by-patient basis [8]. From a urological perspective, the early drainage of urine and prevention of potential infections are essential. Many patients have a combination of severe multiple injuries with unique difficulty in treatment; thus, treatment usually requires comprehensive assessment by multiple specialists from several clinical departments [37].

In our cohort, the overall mortality rate was relatively low at 5.6% (three deaths). These deaths were not directly due to the fact of LUTIs but instead to other associated multiple injuries or complications, such as post-traumatic brain damage, severe infection, and multiple organ failure after surgery. These results are in line with those reported by Spirnak [7], who found that associated urologic injuries are seldom a direct cause of death.

This study has several limitations. One limitation is its retrospective design. Another limitation is the small sample size, which is related to the low incidence of LUTIs in patients with pelvic fractures, especially women. In addition, we did not perform a long-term follow-up, which prevented the assessment of the complications of LUTIs with pelvic fractures.

## 5. Conclusions

According to our data, 7.7% of patients with a pelvic fracture also had an LUTI. The patterns of LUTIs associated with pelvic fractures differ between men and women. In this study, VS-type pelvic fractures were associated with the severity of bladder injury in men. When the pelvis is subjected to VS forces, the risk of bladder injury appears to increase. However, additional studies based on larger sample sizes are needed to further validate our findings. 

## Figures and Tables

**Table 1:** Demographics of the study sample.

Pelvic Fractures with LUTIs Population	
Sex, *n* (%)	
Male	45/54 (83.3%)
Female	9/54 (16.7%)
Age (years), mean ± SD	42.81 ± 14.41
Hospital length of stay (days), mean ± SD	35.37 ± 22.57
ISS, mean ± SD	35.15 ± 14.55
AIS, mean ± SD	25.24 ± 12.17
**Mechanism of injury**	
Road traffic accidents	21/54 (38.9%)
Fall from height	14/54 (25.9%)
Mechanical crush injury	8/54 (14.8%)
Struck by falling objects	7/54 (13.0%)
Truck crush	4/54 (7.4%)
**Whether the fractures were open or closed, *n* (%)**	
Open	10/54 (18.5%)
Closed	44/54 (81.5%)
**Lower urinary tract injury, *n* (%)**	
Isolated bladder injury	23/54 (42.6%)
Isolated urethra injury	23/54 (42.6%)
Bladder + urethra injury	8/54 (14.8%)
**Early complications**	
Pneumonia	24/54 (44.4%)
DVT	23/54 (42.6%)
Wound infection	18/54 (33.3%)
Bacteremia	5/54 (9.3%)
UTI	6/54 (11.1%)
ARDS	13/54 (24.1%)
MODS	6/54 (11.1%)
AKI	8/54 (14.8%)
Shock	25/54 (46.3%)
**Mortality**	3/54 (5.6%)

ISS, injury severity score; AIS, abbreviated injury scale; DVT, deep vein thrombosis; UTI, urinary tract infection; ARDS, acute respiratory distress syndrome; MODS, multiple organ dysfunction syndrome; AKI, acute kidney injury.

**Table 2:** The distribution of pelvic fracture types with LUTIs according to the Tile classification and Young–Burgess classification.

**Group (n)**	**Tile Classification**							***p*-Value**	**Young-Burgess Classification**								***p*-Value**
	B				C				APC				LC			VS	
	1	2	3		1	2	3		I	II	III		I	II	III		
**Patients (54)**																	
Isolated bladder injury	3	6	2		7	4	1	0.172	0	1	4		1	3	4	10	0.994
Isolated urethra injury	2	6	3		8	4	0		1	1	4		0	3	4	10	
Bladder + urethra injury	0	0	2		1	2	3		0	0	1		0	0	2	5	
**Men (45)**																	
Isolated bladder injury	2	4	1		5	3	0	0.265	0	0	4		0	3	2	6	0.965
Isolated urethra injury	2	6	3		8	4	0		1	1	4		0	3	4	10	
Bladder + urethra injury	0	0	2		1	2	2		0	0	1		0	0	2	4	
**Women (9)**																	
Isolated bladder injury	1	2	1		2	1	1	1.000	0	1	0		1	0	2	4	1.000
Isolated urethra injury	0	0	0		0	0	0		0	0	0		0	0	0	0	
Bladder + urethra injury	0	0	0		0	0	1		0	0	0		0	0	0	1	

APC, anteroposterior compression; LC, lateral compression; VS, vertical shear.

**Table 3:** Fracture pattern and injury to the bladder (*n* = 54).

Classification	Subtype	Bladder Injury				*p*-Value
		No.	Contusion	Rupture	Total	
Patients (54)						
Tile	A	0	0	0	0	0.454
	B	11	6	7	24	
	C	13	4	13	30	
Young–Burgess	APC	6	1	5	12	0.342
	LC	7	6	4	17	
	VS	11	3	11	25	

APC, anteroposterior compression; LC, lateral compression; VS, vertical shear.

**Table 4:** Fracture pattern and injury to the urethra (*n* = 54).

Classification	Subtype	Urethra Injury				*p*-Value
		No.	Contusion	Rupture	Total	
Patients (54)						
Tile	A	0	0	0	0	0.879
	B	11	4	9	24	
	C	12	6	12	30	
Young–Burgess	APC	5	5	2	12	0.120
	LC	8	3	6	17	
	VS	10	2	13	25	

APC, anteroposterior compression; LC, lateral compression; VS, vertical shear.

**Table 5:** Fracture pattern and injury to the male and female bladder (*n* = 54).

Classification	Subtype	Bladder Injury				*p*-Value
		No.	Contusion	Rupture	Total	
Men (45)						
Tile	A	0	0	0	0	0.070
	B	11	5	4	20	
	C	13	1	11	25	
Young–Burgess	APC	6	1	4	11	0.037 *
	LC	7	5	2	14	
	VS	11	0	9	20	
**Women (9)**						
Tile	A	0	0	0	0	0.524
	B	0	1	3	4	
	C	0	3	2	5	
Young–Burgess	APC	0	0	1	1	1.000
	LC	0	1	2	3	
	VS	0	3	2	5	

APC, anteroposterior compression; LC, lateral compression; VS, vertical shear. * *p* < 0.05.

**Table 6:** Fracture pattern and injury to the male urethra (*n* = 45).

Classification	Subtype	Urethra Injury				*p*-Value
		No.	Contusion	Rupture	Total	
Men (45)						
Tile	A	0	0	0	0	1.000
	B	7	4	9	20	
	C	8	6	11	25	
Young–Burgess	APC	4	5	2	11	0.148
	LC	5	3	6	14	
	VS	6	2	12	20	

APC, anteroposterior compression; LC, lateral compression; VS, vertical shear.

## Data Availability

Data can be provided upon reasonable request from the corresponding author.
